# Association Between DPPs-4 Inhibitors and Bullous Pemphigoid: Reporting Odds Ratio Analysis Using EudraVigilance Database

**DOI:** 10.3390/ph18121800

**Published:** 2025-11-26

**Authors:** Alex Carbonell Pedrero, Ana Aldea-Perona

**Affiliations:** 1 Faculty of Medicine and Life Sciences, Pompeu Fabra University, 08003 Barcelona, Spain; 2 Hospital del Mar Research Institute, 08003 Barcelona, Spain

**Keywords:** DPP-4 inhibitors, bullous pemphigoid, EudraVigilance

## Abstract

**Background/Objectives:** Bullous pemphigoid (BP) is an autoimmune blistering skin disease. The association between dipeptidyl peptidase 4 inhibitors (DPP-4 inhibitors) and bullous pemphigoid (BP) has been studied in many countries; however, controversy has arisen from analyzing the related risk factors. The objectives of this study are to assess whether the association between DPP-4 inhibitors and bullous pemphigoid in EudraVigilance is statistically significant and to identify the presence of risk factors found in previous studies in a case/exposure group. Our results will be compared with those obtained from the Food and Drug Administration Adverse Event Reporting System database (FAERS). **Methods:** A case/control retrospective observational study was performed using data from the European database EudraVigilance. All reports from 2007 to 2024 (a total of 11,451,738 reports) were gathered and filtered by exposure to DPP-4 inhibitors and development of BP or lack thereof. Association was measured using reporting odds ratios with a 95% confidence interval, and Fisher’s exact test was used to obtain p-values, assuming an alpha error of 0.05. **Results:** The results indicate an association between the consumption of DPP-4 inhibitors and the development of BP (with an odds ratio of 153.5; 95% confidence interval 144.1–163.5; ROR = (a/c)/(b/d); a: 1345 reports of BP associated with DPP-4i; c: 3870 reports of BP associated with other different drugs; b: 25,857 reports of other ADRs and DPP-4i; and d: 11,420,666 reports of ADRs associated with other drugs). The predominant factors in the case/exposure group were male gender (58.6%), age between 65 and 85 years (43.3%), medical history of type 2 diabetes mellitus (30.4%) and consumption of vildagliptin (44.2%). Similar results were found in a prior analysis of the FAERS database (2006–2020). **Conclusions:** This study provides evidence of the association between the consumption of gliptins and the development of BP. Disproportionality measures were estimated to be higher in the exposure group than in the positive controls. As such, BP could appear after several months of exposure, and dermatological monitoring is crucial.

## 1. Introduction

Bullous pemphigoid (BP) is among the most common blistering diseases. Its annual incidence in the general population ranges from 1.5 to 48 cases of BP per 100,000 individuals per year. This increase is most notable among individuals older than 80 years, and has experienced a 2- to 4-fold rise over the past two decades [1,2]. The prevalence of BP ranges from 0.21 to 7.7 per 100,000 individuals per year, and it is highest at extreme ages, reaching ~1/500 among individuals > 90 years [1,2]. BP is generally self-limiting but may persist for years; the 1-year mortality rate has been reported to range from 6% to 40%, underscoring its impact on older adults [3].

The physiopathology of this autoimmune disease stems from the existence of IgG antibodies, which interfere with the function of BP230 and BP180 antigens in the hemidesmosomes of basal keratinocytes [4]. As a result, when hemidesmosomes do not provide a consistent union between the epidermis and dermis, clinical manifestations of the disease can develop, such as subepidermal blisters, pruritus, urticarial plaques and patches [2]. Although the etiology of this autoimmune response is unknown, some cases have reported a pharmacological relation. And, when adverse drug reactions appear, management goes beyond stopping potential trigger drugs; instead, the goal becomes to control inflammation and autoantibody activity while minimizing treatment-related toxicity. High-potency topical corticosteroids are the first-line treatment for localized disease. More extensive or severe cases may require systemic corticosteroids and other immunosuppressants (azathioprine, mycophenolate, methotrexate, ciclosporin, and rituximab). Additional options include anti-inflammatory antibiotics (e.g., tetracyclines and dapsone) and therapies that remove circulating antibodies, such as high-dose intravenous immunoglobulin or plasma exchange [5].

Dipeptidyl peptidase 4 inhibitors (DPP-4i), also known as gliptins, are oral antihyperglycemic drugs used in the pharmacological treatment of type 2 diabetes mellitus (T2DM).

The pharmacological effect of these drugs consists of the inhibition of DPP-4: an enzyme that degrades incretins such as the glucose-dependent insulinotropic polypeptide (GIP) and the glucagon-like peptide-1 (GLP-1). Incretin increases the release of insulin when food reaches the digestive tract to reduce glycemia. In conclusion, gliptins prolong the effect of incretins to better control glycemia and treat T2DM [6].

Although this drug class shares many common features, individual molecules may present differences in their pharmacological properties and adverse effect profiles. For example, vildagliptin has been more frequently linked to elevations in liver enzymes and shows a pharmacovigilance signal for bullous pemphigoid [7]. In addition, saxagliptin and alogliptin have been discussed in relation to the risk of heart failure [8]. By contrast, sitagliptin and linagliptin have demonstrated more consistent tolerability, with linagliptin being particularly suitable for patients with renal impairment due to its predominantly biliary elimination [9,10]. These variations underscore the need for further exploration into the class effects and molecule-specific risks related to rare but clinically relevant adverse events such as bullous pemphigoid.

It has been proposed that this adverse drug reaction may result from DPP-4 inhibition altering the physiological proteolytic degradation of BP180, leading to neo-epitope exposure and immune dysregulation. This causes impaired T-cell function and increased eotaxin release [11], a mechanism that could also explain the long-standing latency between gliptin initiation and the onset of BP. It is suggested that these drugs act as aggravating factors in a background predisposed to immune diseases [12].

Inmunologic and non-immunologic pathways could be involved in the development of BP due to DPP-4i. Vildagliptin has been shown to downregulate key adhesion molecules and may promote IL-6–mediated inflammation, suggesting both structural and inflammatory mechanisms in DBP pathogenesis [13].

Multiple pharmacovigilance studies have reported an association between the consumption of gliptins and the development of BP [7,14,15,16,17,18,19,20,21,22,23,24].

Unfortunately, researchers have not reached an agreement regarding the risk factors associated with this finding. Suspected risk factors in the literature include, but are not limited to, male gender, diagnosis of T2DM, age older than 80 years [25], neurological disorders (stroke, epilepsy, multiple sclerosis, dementia and Parkinson’s disease) [3,11,17] and polypharmacy. This study aims to identify novel data regarding the following points:

(1) More evidence proving the existence of this association on a wider level.
(2) Elucidation of risk factors that play a role in the association between gliptins and BP.
(3) An analysis of whether the population notified by EudraVigilance has or lacks specific characteristics when compared to the findings in the literature.
(4) Exposure of the strengths and weaknesses of the reporting system used by EudraVigilance and propose future modifications.

Therefore, this study was performed based on the following hypothesis and objectives:

**Hypothesis:**  *Exposure to gliptins is associated with a statistically significant increase in reports of BP in the European EudraVigilance database.*

Primary objective: We aim to assess whether the association between gliptins and BP in EudraVigilance is statistically significant.

Secondary objectives are as follows:

- To describe and identify the most prevalent factors in the reports where gliptin is associated with BP (case/exposure group): age, gender, medical history of T2DM2 [9], stroke, epilepsy, multiple sclerosis, dementia and Parkinson’s disease [3,11,17]. These characteristics are presented descriptively, since they have been identified as potential risk factors in previous studies. However, our design does not allow us to confirm such associations.
- To compare our results with the American study performed by Jedlowski et al., which used the Food and Drug Administration (FDA) Adverse Event Reporting System (FAERS) [19].

## 2. Results

### 2.1. Case/Control Analysis

The data requested for the general analysis was provided by EudraVigilance in the form of an Excel spreadsheet. It contained counts of the various conditions explained in the methodology (Section 4.2). In summary, EudraVigilance provided a total of 11,451,738 reports from all ADRs, covering all drugs involved. A total of 5215 of those reports were related to the development of BP in relation to a medicinal product, and specific counts for every type of gliptin, including both positive and negative controls, were also provided.

This analysis showed statistical significance between the consumption of gliptins and the development of BP (ROR 153.5, 95% CI 144.1–163.5). Regarding each gliptin separately, the highest association was found with vildagliptin (ROR 692.9, 95% CI 629.9–762.3), teneligliptin (ROR 394.5, 95% CI 176.4–882.4) and linagliptin (ROR 279.7, 95% CI 250.3–312.5). The lowest association was found with sitagliptin (ROR 37.7, 95% CI 33.6–42.3). No reports were received involving the consumption of anagliptin, gemigliptin or trelagliptin and the development of BP. Very similar results were found with Fisher’s exact test, and *p* < 0.0001 was reached with every gliptin except anagliptin, gemigliptin and trelagliptin (Table 1).

Regarding both positive and negative controls, an ROR of 11.3 (95% CI 9.7–13.2) was obtained from the positive control with furosemide, and an ROR of 0.5 (95% CI 0.3–0.7) was obtained from the negative control with paracetamol.

Reports from the FAERS database identified an ROR higher than one for all gliptins in the EudraVigilance database. Similarly, in the case/control analysis, vildagliptin showed the highest ROR (1022.8, 95% CI 909.45–1150.35), followed by anagliptin, teneligliptin, alogliptin, linagliptin, sitagliptin and saxagliptin [13] (Table 1).

### 2.2. Sensitivity Analysis

Similarly to the results of the case/control analysis, an Excel spreadsheet was provided that contained the counts requested, excluding those that contained any of the aforementioned drugs related to the development of BP.

A higher ROR was found with every gliptin when compared to the ROR of the case/control analysis, except for teneligliptin (ROR 350.1, 95% CI 105.4–1163.1). The highest association was found with vildagliptin (ROR 904.7, 95% CI 816.7–1002.2), followed by teneligliptin (ROR 350.1, 95% CI 105.4–1163.1) and linagliptin (ROR 342.6, 95% CI 304–386.2). No reports were received involving the consumption of anagliptin, gemigliptin or trelagliptin and the development of BP. Very similar results were found with Fisher’s exact test, and *p* < 0.0001 was reached for every gliptin except anagliptin, gemigliptin and trelagliptin (Table 2).

### 2.3. Description of the Case/Exposure Population

A total of 1345 reports were received in which BP was associated with gliptin. In addition, 44.2% of the reports were related to the consumption of vildagliptin.

After filtering the data, a full descriptive analysis was performed on the main presumed risk factors to identify the characteristics of this group (Table 3).

The mean age was 77 years with a standard deviation of 10.6 years and a median of 78 years. In total, 58.6% of the reports were from patients of the male gender, while 34.1% were from the female gender. The most prevalent disease from the medical history of subjects was T2DM, registered in 30.4% of the reports.

Most of the ICSRs were reported by physicians (n = 862; 64%) and other medical professionals (n = 284; 21%), and 72% of ICSRs were reported from non-EU countries. Japan was the country with the highest number of reports (739 reports). All predominant factors identified for the case/exposure group applied to both European Union (EU) and non-EU country reports, but interesting differences were found when evaluating their proportions. EU reports lacked correct age registration in 45.5% of the reports, while non-EU countries lacked this in 28.7% of reports. In non-EU countries, the consumption of vildagliptin was higher (48.8% vs. 39.5%).

Finally, a higher proportion of ADRs notified by physicians was found in EU reports (80.2%) when compared to non-EU countries (59.0%).

The median latency period, defined as the time from the first administration of the DPP-4 inhibitor to the onset of bullous pemphigoid (BP), was 11 months. Given the wide variability and skewed distribution of the data (mean: 19 months; SD: 22.1), the median was considered the most appropriate measure of central tendency. The longest latency was observed for vildagliptin (24.5 months), while the shortest latency was reported for teneligliptin (45 days).

Similarly, the median time from drug withdrawal to the clinical resolution of BP was 33 days. The mean duration was 1.9 months (SD: 2.7), and the large dispersion of values supports the median as a better reflection of the typical course of disease.

The median time between the onset of bullous pemphigoid (BP) and the withdrawal of the DPP-4 inhibitor was 36 days. Again, due to the wide variability and non-normal distribution of the data (mean: 3.5 months; SD: 6.4), the median was considered a more appropriate descriptor of central tendency.

In 11.8% of reports, gliptin was discontinued the same day that BP was diagnosed; in 46.35% of reports, it was retired within a month; and in 6.3% of reports, more than one year passed until gliptin was withdrawn. The longest mean period between the beginning of BP and the withdrawal of gliptin was found for sitagliptin (4.1 months) and vildagliptin (3.6 months), and the shortest period was identified for teneligliptin (1.0 months).

## 3. Discussion

### 3.1. Association Between Gliptins and BP—Case/Control Analysis

The ROR is a disproportionality measure; when its value is greater than 1, this signifies that events have occurred more frequently than expected. However, confirmation of a signal requires validation and expert evaluation, taking into account clinical relevance and other available data. The consistency of results with the biological plausibility of the causal relationship and a positive control supports the robustness of the signal, whereas the absence of disproportionality with a negative control reduces the likelihood of a spurious association. Together with the concordant findings from FAERS reported by Jedlowski et al. [19], these elements strengthen the credibility of the association observed between DPP-4 inhibitors and BP in EudraVigilance. The initial case/control results were expected, as similar results were obtained in the study by Jedlowski et al. [19] for their initial analysis (ROR 109.8 for all gliptins with 95% CI 101.6–118.6) [13]. Jedlowski’s research group performed three different analyses: during the first analysis, all drugs contained in the FAERS database were taken into consideration, whereas in the subsequent studies, only reports in which antihyperglycemic drugs were consumed were included. For these investigations, the factor of having T2DM was excluded from the study. Promising RORs were obtained and were adjusted based on the ROR of both positive and negative controls [13]. This phenomenon highlights the importance of confounding factors and the need to appropriately distinguish between them.

The more significant the association between vildagliptin and the development of BP compared to the other types of gliptins, the broader its use in European clinical practice. This could lead to potential reporting bias and should therefore be interpreted with caution when comparing across individual gliptins. In contrast, findings related to molecules with very few reports, such as teneligliptin (n = 7), were subject to high uncertainty, and specific observations (e.g., shorter latency periods) could not be considered conclusive.

Other pharmacovigilance studies have demonstrated disproportionate reporting for bullous pemphigoid (BP) associated with DPP-4 inhibitors (gliptins), supporting the hypothesis of a potential class effect. However, substantial differences have been identified in the magnitude of the signal, most notably the reporting odds ratio (ROR), which was observed across different databases and regions. In the French pharmacovigilance database (FPVD), Béné et al. (2016) reported a class-level ROR of 67.5, with vildagliptin showing the strongest individual signal (ROR 225.3; 95% CI 148.9–340.9) [24]. For the Spanish database (FEDRA), Molina-Guarneros et al. (2020) produced similar findings with an ROR of 71, with vildagliptin being the most prominent agent [21]. The Japanese (JADER), Arai et al. (2018) and VigiBase data further supported this trend, reporting class-level RORs of 87.6 and 179.4, respectively [20,22].

In the literature, cohort studies differ in that gliptin is more commonly prescribed [14,15]; as such, future observational studies should investigate this subject (see Supplemental Material).

### 3.2. Sensitivity Analysis

The sensitivity analysis was initially conceived to mitigate the lack of stratification between risk factors. From a theoretical perspective, taking into consideration the drugs that were classified as associated with the development of BP, the main idea was to discard all reports in which these drugs could have been suspected.

The higher ROR observed in the sensitivity analysis was expected, given the analytical approach, as the exclusion of reports with other medications reduces reports of other ADRs compared to those of BP. Therefore, this finding should be seen as methodological confirmation that the signal remains robust under stricter analytical conditions, rather than as an indication of increased risk (See Supplemental Material S1 for more information).

### 3.3. Descriptive Analysis

The results, while being simple and free from profound discussion, can support the generation of future hypotheses regarding the risk factors associated with gliptins and BP. Factors such as the age range between 65 and 85 years old, age older than 85 years old, the male gender and the diagnosis of T2DM could be taken into consideration as risk factors for future observational and analytical studies. The most frequently recorded medical history was T2DM (30.4% of reports). As reporting of medical history in spontaneous reports is not mandatory and often incomplete, this proportion should not be interpreted as evidence that gliptins were systematically prescribed for other indications. Any such implication should be treated with caution.

Interestingly, no diagnoses were reported for multiple sclerosis among the gliptin-associated BP reports in our dataset. This finding contrasts with previous studies, which have suggested multiple sclerosis to be a potential risk factor for BP. The absence of such reports in EudraVigilance may reflect multiple factors, including the rarity of this comorbidity in the exposed population, underreporting in spontaneous reporting systems, or differences in population characteristics. Therefore, while our results do not support a link between multiple sclerosis and gliptin-associated BP, this possibility cannot be excluded and warrants further investigation in larger analytical studies.

EudraVigilance was contacted to explain the large number of reports from countries outside of the EU. The main reason for this was that the database not only included reports from the EU, but also gathered reports of serious ADRs from other countries. The large number of reports from Japan could be related to the fact that their treatment of T2DM takes the form of monotherapy with gliptins, while in other countries, gliptins are used in combination with other oral antihyperglycemic drugs [26]. A surprisingly large proportion of missing data was found during the analysis regarding age, country of registration, gender and concomitant medication used. Future changes should be proposed to improve the quality of reports.

### 3.4. Comparison with the FAERS Database Results

In contrast with other pharmacovigilance database studies (Vigibase, FEDRA, FPVD and JADER), the FAERS database yielded an extremely high signal under broad analysis conditions, with an ROR of 109.8 for the DPP-4 inhibitor class, and 1022.8 for vildagliptin. However, this signal declined markedly (ROR = 1.29, non-significant) after restricting the analysis to cases using monotherapy without confounding drugs. When controlling for diabetes as the only indication (a strength of the FAERS analysis), linagliptin emerged as the gliptin with the highest ROR under strict conditions (ROR = 122.3; 95% CI 93.3–159.1), highlighting the influence of analytical strategy on the results.

Despite the differences, similar results were obtained for EudraVigilance when compared to the results from Jedlowski et al. using the FAERS database [19]. Sitagliptin and saxagliptin showed lower disproportionality signals for bullous pemphigoid compared with the other gliptins, whereas vildagliptin demonstrated the highest association. These findings suggest a degree of heterogeneity within the drug classes, although prescription patterns, reporting practices, and pharmacological differences may also contribute to the observed variation.

Drug approval status and methodological heterogeneity could explain some differences between the results from the studies based on pharmacovigilance databases. For example, vildagliptin is not approved in the United States, yet it accounts for a large portion of BP cases in FAERS. This reflects international reporting by manufacturers but also artificially inflates RORs for drugs with few total reports—a known limitation in spontaneous reporting systems. A disproportionality analysis was performed by Huang et al., 2021 [18] between March 2004 and August 2020, only using drugs approved by the FDA (sitagliptin, saxagliptin, linagliptin and alogliptin). The largest disproportionality was found for alogliptin (ROR 94.1; ROR025: 58.3) [18], whereas vildagliptin is more widely used in Europe (e.g., Spain, France, and Greece). This may partly explain why it consistently shows the strongest signal in European datasets.

With respect to methodological heterogeneity, relevant differences were observed across study periods and the number of reports included. In the French Pharmacovigilance Database (FPVD), Béné et al. (2016) analyzed 42 cases reported between 2008 and 2014 [24], while Reolid et al. (2020) examined 22 cases from the Spanish Pharmacovigilance Database (FEDRA) for 2007–2017 [27]. In contrast, Arai et al. (2018) identified 392 cases in the Japanese Pharmacovigilance Database (JADER) covering 2004–2017 [22]. The largest source of data was VigiBase^®^, where 1070 reports of BP associated with DPP-4 inhibitors were collected between 2006 and 2019 [20]. Disproportionality analyses consistently reinforced the same signal, with vildagliptin and sitagliptin being reported more frequently than other agents. Nevertheless, the highest reporting odds ratios (RORs) were observed for teneligliptin (ROR 975; 95% CI: 801.7–1185.9), followed by omarigliptin and vildagliptin. Importantly, only a few studies, such as those based on FAERS and the analysis by Molina-Guarneros et al., adjusted for key confounders such as diabetes indication, concomitant medication, and drug class comparators [9,21].

### 3.5. Future Studies

Using the current results, a more complex project can be planned that analyzes the contribution of each risk factor to the association of BP and the consumption of gliptins. Using the risk factors reported in the literature for the association between the consumption of gliptins and the development of BP, a linear regression model can be created. This statistical tool could be used to determine the weight of each risk factor and the ROR of the association once every risk factor is excluded. RORs can be adjusted to those from both positive and negative controls, such as in Jedlowski’s results [13,19]. The main inconvenience of this suggestion is the great amount of data required, as information for every risk factor would be needed from the 11,451,738 reports that form the full population of this study. To mitigate this technical limitation, AI tools could be used in future studies to facilitate the gathering of the data.

### 3.6. Patient and Physician Recommendations

PB, related to DPP-4 inhibitors, has been detected in post-marketing periods with unknown frequency. A summary of this product’s characteristics recommends discontinuing the drug if PB is suspected. However, even with this advertisement, only 11.8% of the reports removed exposure to the drug on the same day of the diagnosis. Sitagliptin or vildagliptin was only withdrawn after the longest time had passed after the diagnosis of BP. This rare but serious adverse drug reaction requires dermatological monitoring, mainly in patients with risk factors such as type 2 diabetes mellitus. More cautious approaches should be taken when evaluating the possibility of BP associated with sitagliptin or vildagliptin. The latency period between starting DPP-4 inhibitors and the onset of bullous pemphigoid can be prolonged by several months or even years.

Most studies agree on the long latency period that typically elapses between the initiation of DPP-4 inhibitors and the onset of bullous pemphigoid (BP). In most of these studies, the median latency time was approximately 6–8 months, ranging from as short as 10 days to more than 3 years [7,24,25] A recent duration–response analysis by Kridin K et al., 2021 [15] suggested that the highest probability of BP onset occurs 1–2 years after DPP-4 inhibitor initiation, with a median latency of 3.3 years and a continuous, statistically significant risk extending beyond 6 years from treatment initiation. This variability in latency has been documented in various pharmacovigilance databases: 8.5 months in FEDRA (ranged between 1.1 and 68.2 months) [27], 449 days in the Finnish nationwide register (ranged between 1 month and 4 years) [7] and between 6 and 9 months in the FPVD [24]. By contrast, the interval between BP onset and drug discontinuation has not been highlighted in previous studies.

Therefore, patients should be closely monitored for the development of signs or symptoms of this skin condition. More precise information should be provided for the use of these gliptins in order to raise awareness for both patients and physicians.

### 3.7. Limitations

EudraVigilance is designed to support easy signal detection. As for limitations, these signals cannot quantify real risk; therefore, this study requires the support of future, stronger prospective studies [20,28,29]. Although randomized clinical trials are the gold standard for establishing causality, their feasibility in this setting is highly limited. Therefore, robust observational pharmacoepidemiological studies, such as population-based cohort or case–control designs, represent the most suitable approach to further evaluate the association between gliptins and BP.

There is variability in the quality of each report regarding the amount of missing data, which affects the quality of the analysis. Technical limitations were also found for the gathering of secondary variables from the control and non-exposure groups, which limited the scope of our research.

Our decision to focus on reports where gliptins were considered the only suspected drugs was based on the risk of obtaining incomplete information for concomitant medications in spontaneous reporting systems. This restrictive approach enhances interpretability and reduces confounding, and it has also been applied in previous pharmacovigilance studies, such as that by Jedlowski et al. They demonstrated that the signal for DPP-4 inhibitors and BP remained significant when restricted to single-agent cases [19].

Due to the inherent limitations of spontaneous reporting systems, population denominators and comparator groups are not available, which prevented the use of multivariable models such as logistic regression. Therefore, our analysis was restricted to a descriptive characterization of BP reported with gliptins.

## 4. Materials and Methods

This is a case/control retrospective observational study using data from the European database EudraVigilance (European Medicines Agency, 1083 HS Amsterdam, The Netherlands. Data extracted: December 2024). This option was selected to enable a comparison with the FAERS results published by Jedlowski et al. [19]. This article fulfills the 2024 Declaration of Helsinki—Ethical Principles for Medical Research Involving Human Participants—developed by the World Medical Association (WMA). The current study was approved by the Ethics Committee of Hospital del Mar in Barcelona.

### 4.1. Database Source

All information was extracted from the European EudraVigilance: an extensive pharmacovigilance database which gathers reports of adverse drug reactions caused by medicines authorized in the European Economic Area (EEA). This information is displayed as individual case safety reports (ICSRs) [30].

This database is managed by the European Medicines Agency (EMA) and enables the sharing of ICSRs with researchers, competent national authorities, marketing authorization holders and the public. The EMA publishes an annual report summarizing the activities of EudraVigilance [30]. The aggregated data from this database is accessible to the public and free of charge. Researchers, competent national authorities and marketing authorization holders can obtain additional information by following specific directives designed for each level [30]. A request for access to level 2A information was performed, which included an expanded set of anonymized ICSRs submitted to EudraVigilance for pharmacovigilance purposes. Further measures were taken to ensure the correct use of the data, such as the signing of a confidentiality agreement.

The anonymity of the data was respected, and the results of the search were coded with a registration number for each report. Once obtained, the data was filtered by the suspected drug responsible for the drug adverse reaction. Only in those reports in which a duplicate registration number was found did we contact EudraVigilance to ensure that it was a duplicate and excluded the duplicated report.

### 4.2. Population

The population is a combination of reports reported to the EudraVigilance database from April 2007, as the first instance of gliptin (sitagliptin Januvia^®^) [31] was approved in the European Union (EU) in 2007 to April 2024. Cases were defined as reports with an adverse drug reaction classified as “Pemphigoid” by the Medical Dictionary for Regulatory Activities (MedDRA^®^), and controls were defined as reports with an adverse drug reaction not classified as “Pemphigoid”. Exposure refers to reports that included exposure to gliptins, while non-exposure refers to reports that did not include exposure to gliptins. The use of positive and negative control groups contextualized the results. Positive controls were ascertained from reports that included the prescription of furosemide: a drug known to be associated with BP and used as a positive control in similar studies [11,19,24]. Negative controls were obtained by reports that included the prescription of paracetamol (acetaminophen) [19,24].

The content obtained from EudraVigilance regarding the primary objective included the total number of reports sent to EudraVigilance for each of the following drugs (with D representing one of the following three): (1) gliptin, (2) furosemide or (3) paracetamol. Reports were obtained for adverse drug reaction combinations (with E representing bullous pemphigoid): reports that include E associated with D, reports that include E regardless of whether D is included and reports that include D regardless of whether E is included.

To achieve the secondary objective, an additional set of information was requested to analyze the presence of secondary variables. Individual case safety reports (ICSRs), including all the secondary variables displayed in this protocol, were needed for reports related to the development of bullous pemphigoid associated with the consumption of each drug: (1) gliptin, (2) paracetamol, and (3) furosemide.

Exclusion criteria are as follows: reports including exposure to more than one gliptin, reports including a combined exposure to gliptin and any other antihyperglycemic drug, reports including a medical history of bullous pemphigoid prior to the adverse drug reaction, and duplicated reports.

### 4.3. Variables

- Primary variables: Adverse drug reactions (ADRs) and the suspected drug.
- Secondary variables: Age, gender, medical history of type 2 diabetes mellitus, medical history of stroke, medical history of epilepsy, medical history of multiple sclerosis, medical history of dementia, medical history of Parkinson’s disease, other medical histories, year in which each disease was diagnosed, concomitant medication, start and end dates of the pharmacological prescription and adverse drug reaction, number of notified drugs before the adverse drug reaction, latency period (time between the start of drug administration and the start of the adverse drug reaction), and other adverse drug reactions notified in the same report.

### 4.4. Statistical Analysis

The analysis was performed using disproportionality measures. This is a well-known method used in pharmacovigilance studies that detects signals. The statistical tool was the reporting odds ratio (ROR) with a confidence interval of 95% (95% CI). This method uses the quotient between the odds of exposure in the case group and the odds of exposure in the control group [28,30]. ROR = (a/c)/(b/d); a: number of BP reports associated with DPP-4i; c: number of BP reports associated with other drugs; b: number of reports containing a reference to any other ADR and DPP-4i; d: number of reports referring to ADRs associated with other drugs. Additionally, Fisher’s exact test was performed to determine *p*-values, assuming an alpha error of 0.05. The statistical analysis was carried out using Excel and R version 4.4.0.

### 4.5. Sensitivity Analysis

Many drugs have been associated with the development of bullous pemphigoid in the literature. Taking this factor into consideration, an additional sensitivity disproportionality analysis was performed. This analysis excluded all reports associated with the prescription of drugs known to cause the development of BP: aspirin, D-penicillamine, enalapril, erlotinib, etanercept, everolimus, furosemide, ibuprofen, levofloxacin, nivolumab, pembrolizumab, phenacetin, psoralens with UVA, rifampicin, serratiopeptidase, sirolimus and tetanus toxoid [11] (see the Supplementary Materials for further information.)

## 5. Conclusions

This study supports the evidence that there is an association between the consumption of gliptins and the development of BP. The estimated disproportionality measures were higher than positive controls, with the most predominant risk factors in the case/exposure group being male gender, an age between 65 and 85 years, consumption of vildagliptin and a medical history of T2DM. Similar results were obtained when comparing these findings to the FAERS analysis performed by Jedlowski et al. Reactions could appear after several months of exposure, making dermatological monitoring crucial for the detection of BP and the immediate removal of DPP-4 inhibitors.

## Figures and Tables

**Table 1:** Reporting odds ratio (ROR) for the association between different dipeptidyl peptidase-4 inhibitors and Bullous.

Drug	Reports of BP Associated with DPP-4i (A)	Reports of Other ADR Associated with DPP-4i (B)	ROR (95% CI)	Fisher’s Exact Test (95% CI)	ROR (95% CI) Jedlowski [19]
All gliptins	1345	27,202	153.5 (144.1–163.5)	154.2 (144.5–164.8)	109.79 (101.61–118.62)
Vildagliptin	595	2722	692.9 (629.9–762.3)	688.8 (628.9–785.7)	1022.83 (909.45–1150.35)
Teneligliptin	7	46	394.5 (176.4–882.4)	397.2 (149.3–905.3)	521.87 (311.10–875.43)
Linagliptin	373	3525	279.7 (250.3–312.5)	279.6 (249.8–315.7)	116.91 (100.25–136.35)
Alogliptin	34	554	144.4 (102–204.6)	144 (99.1–205.8)	162.71 (125.81–210.43)
Saxagliptin	26	885	66.8 (45.1–98.7)	66.7 (43.3–98.7)	11.17 (6.00–20.80)
Sitagliptin	310	19,464	37.7 (33.6–42.3)	37.7 (33.5–42.3)	33.42 (28.99–38.52)
Gemigliptin	0	2	0	0	-
Anagliptin	0	4	0	0	628.63 (221.36–1785.24)
Trelagliptin	0	0	0	0	-

A: number of BP reports related to each respective drug. B: number of ADR reports related to each respective drug. ROR: reporting odds ratio. CI: confidence interval. BP: bullous pemphigoid. ADR: adverse drug reaction.

**Table 2:** Sensitivity analysis results.

Drug	Reports of BP Associated with DPP-4i (A)	Reports of Other ADR Associated with DPPP-4i (B)	ROR (95% CI)	Fisher’s Exact Test (95% CI)
All gliptins	1197	25,685	203.8 (189.9–218.6)	203.4 (190.7–218.1)
Vildagliptin	531	2455	904.7 (816.7–1002.2)	909.2 (796.3–1009.4)
Teneligliptin	3	27	350.1 (105.4–1163.1)	350.7 (67.5–1122.8)
Linagliptin	324	3229	342.6 (304–386.2)	343.0 (300.4–390.2)
Alogliptin	33	502	198.7 (139.4–283.2)	198.4 (134.5–285.5)
Saxagliptin	25	815	89.2 (59.8–132.9)	89.2 (57.3–132.5)
Sitagliptin	281	18,651	46.3 (41–52.4)	46.3 (40.8–52.4)
Gemigliptin	0	2	0	0
Anagliptin	0	4	0	0
Trelagliptin	0	0	0	0

A: number of reports of BP related to each respective drug. B: number of reports of any ADR related to each respective drug. ROR: reporting odds ratio. CI: confidence interval. BP: bullous pemphigoid. ADR: adverse drug reaction.

**Table 3:** Description of the case/exposure group.

Type of Risk Factor	Risk Factor	Count	Percentage
Age	Younger than 65 years old	103	7.6%
	Between 65 and 85 years old	582	43.3%
	Older than 85 years old	238	17.7%
	Unspecified	422	31.4%
Gender	Male	788	58.6%
	Female	459	34.1%
	Unspecified	98	7.3%
Medical history	T2DM	409	30.4%
	Stroke	83	6.2%
	Dementia	7	0.5%
	Parkinson	7	0.5%
	Epilepsy	4	0.3%
	Multiple sclerosis	0	0%
Type of gliptin	Vildagliptin	594	44.2%
	Linagliptin	372	27.6%
	Sitagliptin	309	23%
	Alogliptin	34	2.5%
	Saxagliptin	26	1.9%
	Teneligliptin	7	0.5%
	Unspecified	3	0.2%

## Data Availability

The data of this research were obtained from the EudraVigilance Database: https://www.adrreports.eu/en/data_source.html (accessed on 12 December 2024). This website gives access to web reports on suspected side effects. Each time you search for a web report, you will be shown a disclaimer. To view individual reports, you must confirm that you have read and understood the disclaimer.

## Supplementary Materials

The following supporting information can be downloaded at https://www.mdpi.com/article/10.3390/ph18121800/s1, Supplementary Materials S1. It includes the ROR formula; Reporting Odds Ratio calculations of all DPP-4 inhibitors and the positive and negative controls; all t2 × 2 tables and a List of active substance excluded form the sensitivity analysis.

## Abbreviations

The following abbreviations are used in this manuscript:

ADR	Adverse drug reaction
BP	Bullous pemphigoid
CI	Confidence interval
DPP-4i	Dipeptidyl peptidase 4 inhibitors
EMA	European Medicines Agency
EU	European Union
FAERS	FDA Adverse Event Reporting System (FAERS) database
FDA	Food and Drugs Agency
FEDRA	Farmacovigilancia Española, Datos de Reacciones Adversas
FPVD	Franch Pharmacovigilance Database
GLP-1	Glucagon-like peptide-1
ICSR	Individual Case Safety Report
JADER	Japanese Adverse Drug Event Report
MedDRA	Medical Dictionary for Regulatory Activities
ROR	Reporting odds ratio
T2DM	Type 2 diabetes mellitus
