# Supplementary material for: Association Between DPPs-4 Inhibitors and Bullous Pemphigoid: Reporting Odds Ratio Analysis Using EudraVigilance Database
Source: Pharmaceuticals (Basel). 2025 Nov 26;18(12):1800. doi: 10.3390/ph18121800 (PMC12735842; doi:10.3390/ph18121800)
Supplement: Supplementary file 1 [file pharmaceuticals-18-01800-s001.zip › pharmaceuticals-3814997-supplementary.pdf]

Supplemental Material S1

Formulas of the Reporting Odds Ratio (ROR):

First of all, the results are organized into a 2×2 table.

	Pemphigoid	No pemphigoid	
Exposition	a	b	a+b
Non-exposition	c	d	c+d
	a+c	b+d	a+b+c+d

ROR is calculated by using one of the following formulas: $\frac{a/c}{b/d}$ or ad/bc

Confidence intervals (CI) are calculated using the following formula:

$$CI\ 95\% = ROR \cdot\ e^{\pm 1.96 \cdot \sqrt{\frac{1}{a}+\frac{1}{b}+\frac{1}{c}+\frac{1}{d}}}$$

Table S1. Reporting Odds Ratio of all DPP4 inhibitors and the positive and negative controls

	Vildagliptin	Sitagliptin	Saxagliptin	Linagliptin	Alogliptin	Anagliptin	Gemigliptin	Teneligliptin	Trelagliptin	All DPP4-inhibitors (Total)	Furosemide	Paracetamol
**Count A**	595	310	26	373	34	0	0	7	0	1345	175	27
**Count A+B**	2722	19464	885	3525	554	4	2	46	0	27202	35150	124556
**Count A+C**	5215											
**Count A+B+C+D**	11451738											
**ROR**	692.95	37.7	66.76	279.67	144.45			394.49		153.5	11.33	0.47
**IC 95%**	629.91–762.28	33.59–42.33	45.15–98.72	250.3–312.49	101.99–204.58			176.37–882.36		144.1–163.52	9.74–13.18	0.324–0.69

DPP4-inhibitors: Dipeptidyl peptidase 4 inhibitors; ROR: Reporting Odds Ratio; A: Notifications of the association Pemphigoid and the suspected drug (exposition); B: Notifications with the association of the drug with any other Adverse drug reaction. C: Notifications of pemphigoid caused by another drug. D: Notifications of ADRs associated with other drugs.

Table S2. 2×2 table for Rerpoting Odds Ratio calculations with Vidagliptin

		Pemphigoid		
		YES	NO	
Vildagliptin	YES	595	2127	2722
	NO	4620	11444396	11449016
		5215	11446523	11451738

Table S3. 2×2 table for Reporting Odds Ratio calculations with Sitagliptin

		Pemphigoid		
		YES	NO	
Sitagliptin	YES	310	19154	19464
	NO	4905	11427369	11432274
		5215	11446523	11451738

Table S4. 2×2 table for Reporting Odds Ratio calculations with Saxaglitpin

		Pemphigoid		
		YES	NO	
Saxagliptin	YES	26	859	885
	NO	5189	11445664	11450853
		5215	11446523	11451738

Table S5. 2×2 table for Reporting Odds Ratio calculations with Linagliptin

		Pemphigoid		
		YES	NO	
Linagliptin	YES	373	3152	3525
	NO	4842	11443371	11448213
		5215	11446523	11451738

Table S6. 2×2 table for Reporting Odds Ratio calculations with Alogliptin

		Pemphigoid		
		YES	NO	
Alogliptin	YES	34	520	554
	NO	5181	11446003	11451184
		5215	11446523	11451738

Table S7. 2×2 table for Reporting Odds Ratio calculations with Anagliptin

		Pemphigoid		
		YES	NO	
Anagliptin	YES	0	4	4
	NO	5215	11446519	11451734
		5215	11446523	11451738

Table S8. 2×2 table for Reporting Odds Ratio calculations with Gemigliptin

		Pemphigoid		
		YES	NO	
Gemigliptin	YES	0	2	2
	NO	5215	11446521	11451736
		5215	11446523	11451738

Table S9. 2×2 table for Reporting Odds Ratio calculations with Tenegliptin

		Pemphigoid		
		YES	NO	
Teneligliptin	YES	7	39	46
	NO	5208	11446484	11451692
		5215	11446523	11451738

Table S10. 2×2 table for Reporting Odds Ratio calculations with Trelagliptin

		Pemphigoid		
		YES	NO	
Trelagliptin	YES	0	0	0
	NO	5215	11446523	11451738
		5215	11446523	11451738

Table S11. 2×2 table for Reporting Odds Ratio calculations with total DPP4 inhibitors

		Pemphigoid		
		YES	NO	
DPP-4i	YES	1345	25857	27202
	NO	3870	11420666	11424536
		5215	11446523	11451738

Table S12. 2×2 table for Reporting Odds Ratio calculations with Furosemide

		Pemphigoid		
		YES	NO	
Furosemide	YES	175	34975	35150
	NO	5040	11411548	11416588
		5215	11446523	11451738

Table S13. 2×2 table for Reporting Odds Ratio calculations with Paracetamol

		Pemphigoid		
		YES	NO	
Paracetamol	YES	27	124529	124556
	NO	5188	11321994	11327182
		5215	11446523	11451738

Sensitivity analysis

Table S14. Reporting Odds Ratio of all DPP4 inhibitors and the positive and negative controls

	Vildagliptin	Sitagliptin	Saxagliptin	Linagliptin	Alogliptin	Anagliptin	Gemigliptin	Teneligliptin	Trelagliptin	All DPP4-inhibitors (Total)
Count A	531	281	25	324	33	0	0	3	0	1197
Count A+B	2455	18651	815	3229	502	4	2	27	0	25685
Count A+C	3624									
Count A+B+C+D	10144922									
ROR	904,73	46,32	89,16	342,65	198,7			350,08		203,76
IC 95%	629.91-762.28	33.59-42.33	45.15-98.72	250.3-312.49	101.99 - 204.58			176.37-8802.36		144.1-163.52

DPP4-inhibitors: Dipeptidyl peptidase 4 inhibitors; ROR: Reporting Odds Ratio; A: Notifications of the association Pemphigoid and the suspected drug (exposition); B: Notifications with the association of the drug with any other Adverse drug reaction. C: Notifications of pemphigoid caused by another drug. D: Notifications of ADRs associated with other drugs.

Table S15. 2×2 table for Reporting Odds Ratio calculations for all DPP4-inhibitors

		Pemphigoid		
		YES	NO	
DPP-4i	YES	1197	24488	25685
	NO	2427	10116810	10119237
		3624	10141298	10144922

Table S16. 2×2 table for Reporting Odds Ratio calculations with Vildagliptin

		Pemphigoid		
		YES	NO	
Vildagliptin	YES	531	1924	2455
	NO	3093	10139374	10142467
		3624	10141298	10144922

Table S17. 2×2 table for Reporting Odds Ratio calculations with Sitagliptin

		Pemphigoid		
		YES	NO	
Sitagliptin	YES	281	18370	18651
	NO	3343	10122928	10126271
		3624	10141298	10144922

Table S18. 2×2 table for Reporting Odds Ratio calculations with Saxagliptin

		Pemphigoid		
		YES	NO	
Saxagliptin	YES	25	790	815
	NO	3599	10140508	10144107
		3624	10141298	10144922

Table S19. 2×2 table for Reporting Odds Ratio calculations with Linagliptin

		Pemphigoid		
		YES	NO	
Linagliptin	YES	324	2905	3229
	NO	3300	10138393	10141693
		3624	10141298	10144922

Table S20. 2×2 table for Reporting Odds Ratio calculations with Alogliptin

		Pemphigoid		
		YES	NO	
Alogliptin	YES	33	469	502
	NO	3591	10140829	10144420
		3624	10141298	10144922

Table S21. 2×2 table for Reporting Odds Ratio calculations with Anagliptin

		Pemphigoid		
		YES	NO	
Anagliptin	YES	0	4	4
	NO	3624	10141294	10144918
		3624	10141298	10144922

Table S22. 2×2 table for Reporting Odds Ratio calculations with Gemigliptin

		Pemphigoid		
		YES	NO	
Gemigliptin	YES	0	2	2
	NO	5215	10141296	10146511
		3624	10141298	10144922

Table S23. 2×2 table for Reporting Odds Ratio calculations with Tenegliptin

		Pemphigoid		
		YES	NO	
Teneligliptin	YES	3	24	27
	NO	3621	10141274	10144895
		3624	10141298	10144922

Table S24. 2×2 table for Reporting Odds Ratio calculations with Trelagliptin

		Pemphigoid		
		YES	NO	
Trelagliptin	YES	0	0	0
	NO	5215	11446523	11451738
		3624	10141298	10144922

List of active substance considered in the sensitivity analysis.

**Drug** Verheyden, M.J.; Bilgic, A.; Murrell, D.F. A Systematic Review of Drug-Induced Pemphigoid. *Acta Derm. Venereol.* **2020**, *100*(1), adv00224. https://doi.org/10.2340/00015555-3457.	**Active Substance (High Level)**
aspirin	5-ACETYLSALICYLIC ACID
	ACETYLSALICYLIC ACID
	CAFFEINE CITRATE, ASPIRIN BP, PARACETAMOL PH. EUR.
	DEXTROMETHORPHAN HYDROBROMIDE, ACETAMINOPHEN MIXTURE WITH ASPIRIN
	METOCLOPRAMIDE, LYSINE ASPIRIN
D-penicillamine	PENICILLAMINE
	PENICILLAMINE, LACTOSE MONOHYDRATE, POTATO STARCH, POVIDONE, TALC
enalapril	AMLODIPINE, ENALAPRIL
	ENALAPRIL
erlotinib	ERLOTINIB
etanercept	ETANERCEPT, HYDROCHLOROTHIAZIDE
	ETANERCEPT
everolimus	EVEROLIMUS
furosemide	AMILORIDE HYDROCHLORIDE, FUROSEMIDE
	FUROSEMIDE
ibuprofen	ASCORBIC ACID, IBUPROFEN
	CHLORPHENAMINE MALEATE, IBUPROFEN
	CHLORPHENAMINE MALEATE, PHENYLEPHRINE HYDROCHLORIDE, IBUPROFEN
	CHLORPHENAMINE MALEATE, PSEUDOEPHEDRINE HYDROCHLORIDE, IBUPROFEN
	CHLORZOXAZONE, IBUPROFEN
	DEXIBUPROFEN
	DIPHENHYDRAMINE HYDROCHLORIDE, IBUPROFEN
	ERGOTAMINE TARTRATE, IBUPROFEN, ANHYDROUS CAFFEINE
	FAMOTIDINE, IBUPROFEN

	GLUCOSAMINE SULFATE, IBUPROFEN, CHONDROITIN SULFATE SODIUM
	HOMATROPINE METHYLBROMIDE, IBUPROFEN
	HYDROCODONE BITARTRATE, IBUPROFEN
	IBUPROFEN
	IBUPROFEN, ACETYLSALICYLIC ACID
	IBUPROFEN, CAFFEINE
	IBUPROFEN, CODEINE
	IBUPROFEN, HEPARINOID
	IBUPROFEN, HYOSCINE BUTYLBROMIDE
	IBUPROFEN, ISOPROPYL MYRISTATE
	IBUPROFEN, LEVOMENTHOL
	IBUPROFEN, MALTITOL
	IBUPROFEN, MENTHOL
	IBUPROFEN, METHOCARBAMOL
	IBUPROFEN, PARACETAMOL
	IBUPROFEN, PARACETAMOL, CODEINE PHOSPHATE
	IBUPROFEN, PSEUDOEPHEDRINE
	OXYCODONE HYDROCHLORIDE, IBUPROFEN
	PHENYLEPHRINE HYDROCHLORIDE, IBUPROFEN
	PITOFENONE HYDROCHLORIDE, FENPIVERINIUM BROMIDE, IBUPROFEN
	PSEUDOEPHEDRINE HYDROCHLORIDE, IBUPROFEN BP
levofloxacin	AMOXICILLIN TRIHYDRATE, LANSOPRAZOLE, LEVOFLOXACIN HEMIHYDRATE
	LEVOFLOXACIN
	DEXAMETHASONE, LEVOFLOXACIN
nivolumab	NIVOLUMAB
	NIVOLUMAB, RELATLIMAB
pembrolizumab	PEMBROLIZUMAB
	PEMBROLIZUMAB, FAVEZELIMAB
	PEMBROLIZUMAB, QUAVONLIMAB
	PEMBROLIZUMAB, VIBOSTOLIMAB
phenacetine	AMINOPHENAZONE, PHENACETIN, CAFFEINE
	AMINOPHENAZONE, PHENACETIN, ETHYLMORPHINE HYDROCHLORIDE
	AMINOPHENAZONE, PHENACETIN, SODIUM SALICYLATE, CAFFEINE
	PHENACETIN
	PHENACETIN, ACETANILIDE, ACETYLSALICYLIC ACID, CAFFEINE
	PHENACETIN, ACETYLSALICYLIC ACID, CAFFEINE
	PHENACETIN, PHENOBARBITAL, ACETYLSALICYLIC ACID, CAFFEINE, CODEINE
psoralenes with UVA	PSORALEN
rifampicin	ISONIAZID, PYRAZINAMIDE, RIFAMPICIN
	ISONIAZID, PYRAZINAMIDE, RIFAMPICIN, ETHAMBUTOL HYDROCHLORIDE
	ISONIAZID, RIFAMPICIN

	RIFAMPICIN
	RIFAMPICIN, TRIMETHOPRIM
serratiopeptidase	SERRAPEPTASE
sirolimus	SIROLIMUS
	SIROLIMUS ALBUMIN-BOUND
tetanic toxoid	ALUMINIUM PHOSPHATE, ALUMINIUM, DIPHTHERIA TOXOID ADSORBED ON ALUMINIUM HYDROXIDE, HYDRATED AND ALUMINIUM PHOSPHATE, TETANUS TOXOID ADSORBED ON ALUMINIUM HYDROXIDE, HYDRATED AND ALUMINIUM PHOSPHATE, PERTUSSIS TOXOID ADSORBED ON ALUMINIUM HYDROXIDE, HYDRATED AND ALUMINIUM PHOSPHATE, PERTUSSIS PERTACTIN ADSORBED ON ALUMINIUM HYDROXIDE, HYDRATED AND ALUMINIUM PHOSPHATE, PERTUSSIS FILAMENTOUS HAEMAGGLUTININ ON ALUMINIUM HYDROXIDE, HYDRATED AND ALUMINIUM PHOSPHATE
	ALUMINIUM PHOSPHATE, DIPHTHERIA TOXOID ADSORBED ON ALUMINIUM HYDROXIDE, HYDRATED AND ALUMINIUM PHOSPHATE, TETANUS TOXOID ADSORBED ON ALUMINIUM HYDROXIDE, HYDRATED AND ALUMINIUM PHOSPHATE, PERTUSSIS TOXOID ADSORBED ON ALUMINIUM HYDROXIDE, HYDRATED AND ALUMINIUM PHOSPHATE, PERTUSSIS PERTACTIN ADSORBED ON ALUMINIUM HYDROXIDE, HYDRATED AND ALUMINIUM PHOSPHATE, PERTUSSIS FILAMENTOUS HAEMAGGLUTININ, ALGELDRATE
	ALUMINIUM PHOSPHATE, DIPHTHERIA TOXOID ADSORBED ON ALUMINIUM PHOSPHATE, TETANUS TOXOID ADSORBED ON ALUMINIUM PHOSPHATE, BORDETELLA PERTUSSIS TOXOID ADSORBED ON ALUMINIUM PHOSPHATE, BORDETELLA PERTUSSIS FILAMENTOUS HAEMAGGLUTININ ADSORBED ON ALUMINIUM PHOSPHATE, BORDETELLA PERTUSSIS PERTACTIN ADSORBED ON ALUMINIUM PHOSPHATE, BORDETELLA PERTUSSIS FIMBRIAE TYPE 2 AND 3 ADSORBED ON ALUMINIUM PHOSPHATE, POLIOVIRUS (INACTIVATED) TYPE 1 (MAHONEY STRAIN) PRODUCED ON VERO CELLS, POLIOVIRUS (INACTIVATED) TYPE 2 (MEF-1 STRAIN) PRODUCED ON VERO CELLS, POLIOVIRUS (INACTIVATED) TYPE 3 (SAUKETT STRAIN) PRODUCED ON VERO CELLS
	ALUMINIUM PHOSPHATE, DIPHTHERIA TOXOID ADSORBED ON ALUMINIUM PHOSPHATE, TETANUS TOXOID ADSORBED ON ALUMINIUM PHOSPHATE, BORDETELLA PERTUSSIS TOXOID ADSORBED ON ALUMINIUM PHOSPHATE, BORDETELLA PERTUSSIS FILAMENTOUS HAEMAGGLUTININ ADSORBED ON ALUMINIUM PHOSPHATE, BORDETELLA PERTUSSIS PERTACTIN ADSORBED ON ALUMINIUM PHOSPHATE, PERTUSSIS FIMBRIAL AGGLUTINOGENS (FIM) 2 AND 3
	ALUMINIUM PHOSPHATE, DIPHTHERIA TOXOID, TETANUS TOXOID, HEPATITIS B SURFACE ANTIGEN, PERTUSSIS TOXOID, PERTUSSIS PERTACTIN, POLIOVIRUS (INACTIVATED) TYPE 3 (SAUKETT STRAIN), POLIOVIRUS (INACTIVATED) TYPE 2 (MEF-1

	STRAIN), POLIOVIRUS (INACTIVATED) TYPE 1 (MAHONEY STRAIN), ALGELDRATE, FILAMENTOUS HAEMAGGLUTININ
	ALUMINIUM PHOSPHATE, HAEMOPHILUS INFLUENZAE TYPE B POLYRIBOSYLRIBITOL PHOSPHATE CONJUGATED TO MENINGOCOCCAL PROTEIN ADSORBED ON AMORPHOUS ALUMINIUM HYDROXYPHOSPHATE SULFATE, DIPHTHERIA TOXOID ADSORBED ON ALUMINIUM PHOSPHATE, TETANUS TOXOID ADSORBED ON ALUMINIUM PHOSPHATE, BORDETELLA PERTUSSIS TOXOID ADSORBED ON ALUMINIUM PHOSPHATE, BORDETELLA PERTUSSIS FILAMENTOUS HAEMAGGLUTININ ADSORBED ON ALUMINIUM PHOSPHATE, BORDETELLA PERTUSSIS PERTACTIN ADSORBED ON ALUMINIUM PHOSPHATE, BORDETELLA PERTUSSIS FIMBRIAE TYPE 2 ADSORBED ON ALUMINIUM PHOSPHATE, BORDETELLA PERTUSSIS FIMBRIAE TYPE 3 ADSORBED ON ALUMINIUM PHOSPHATE, HEPATITIS B SURFACE ANTIGEN (RDNA) ADSORBED ON AMORPHOUS ALUMINIUM HYDROXYPHOSPHATE SULPHATE [PRODUCED IN S. CEREVISIAE BY RDNA], POLIOVIRUS (INACTIVATED) TYPE 1 (MAHONEY STRAIN) PRODUCED ON VERO CELLS, POLIOVIRUS (INACTIVATED) TYPE 2 (MEF-1 STRAIN) PRODUCED ON VERO CELLS, POLIOVIRUS (INACTIVATED) TYPE 3 (SAUKETT STRAIN) PRODUCED ON VERO CELLS, AMORPHOUS ALUMINIUM HYDROXYPHOSPHA
	ALUMINIUM PHOSPHATE, POLIOVIRUS (INACTIVATED) TYPE 3 (SAUKETT STRAIN), PERTUSSIS FILAMENTOUS HAEMAGGLUTININ ADSORBED ON ALUMINIUM HYDROXIDE, HYDRATED, POLIOVIRUS (INACTIVATED) TYPE 2 (MEF-1 STRAIN), PERTUSSIS TOXOID ADSORBED ON ALUMINIUM HYDROXIDE, HYDRATED, TETANUS TOXOID ADSORBED ON ALUMINIUM HYDROXIDE, HYDRATED, POLIOVIRUS (INACTIVATED) TYPE 1 (MAHONEY STRAIN), PERTUSSIS PERTACTIN ADSORBED ON ALUMINIUM HYDROXIDE, HYDRATED, HAEMOPHILUS TYPE B POLYSACCHARIDE CONJUGATED TO TETANUS TOXOID ADSORBED ON ALUMINIUM PHOSPHATE, HEPATITIS B SURFACE ANTIGEN (RDNA) ADSORBED ON ALUMINIUM PHOSPHATE [PRODUCED IN S. CEREVISAE CELLS BY RDNA], DIPHTHERIA TOXOID ADSORBED ON ALUMINIUM HYDROXIDE, HYDRATED, ALGELDRATE
	ALUMINIUM PHOSPHATE, TETANUS TOXOID ADSORBED ON ALUMINIUM PHOSPHATE
	DIPHTHERIA AND TETANUS TOXOIDS AND PERTUSSIS, HAEMOPHILUS INFLUENZAE TYPE B (HIB), HEPATITIS B SURFACE ANTIGEN
	DIPHTHERIA TOXOID, TETANUS TOXOID, HAEMOPHILUS TYPE B CONJUGATE VACCINE (TETANUS TOXOID CONJUGATE), HEPATITIS B SURFACE ANTIGEN (RDNA), POLIOVIRUS (INACTIVATED) TYPE 3 (SAUKETT STRAIN), PERTUSSIS FILAMENTOUS HAEMAGGLUTININ ADSORBED ON ALUMINIUM HYDROXIDE, HYDRATED, POLIOVIRUS (INACTIVATED) TYPE 2 (MEF-1 STRAIN), PERTUSSIS TOXOID

	ADSORBED ON ALUMINIUM HYDROXIDE, HYDRATED, POLIOVIRUS (INACTIVATED) TYPE 1 (MAHONEY STRAIN), ALGELDRATE, TETANUS PROTEIN
	DIPHTHERIA TOXOID, TETANUS TOXOID, HAEMOPHILUS TYPE B CONJUGATE VACCINE (TETANUS TOXOID CONJUGATE), HEPATITIS B SURFACE ANTIGEN, POLIOVIRUS (INACTIVATED) TYPE 3 (SAUKETT STRAIN), PERTUSSIS FILAMENTOUS HAEMAGGLUTININ ADSORBED ON ALUMINIUM HYDROXIDE, HYDRATED, POLIOVIRUS (INACTIVATED) TYPE 2 (MEF-1 STRAIN), PERTUSSIS TOXOID ADSORBED ON ALUMINIUM HYDROXIDE, HYDRATED, POLIOVIRUS (INACTIVATED) TYPE 1 (MAHONEY STRAIN), ALGELDRATE, TETANUS PROTEIN
	DIPHTHERIA TOXOID, TETANUS TOXOID, HAEMOPHILUS TYPE B CONJUGATE VACCINE (TETANUS TOXOID CONJUGATE), PERTUSSIS TOXOID, PERTUSSIS FILAMENTOUS HAEMAGGLUTININ, POLIOVIRUS (INACTIVATED) TYPE 3 (SAUKETT STRAIN), POLIOVIRUS (INACTIVATED) TYPE 2 (MEF-1 STRAIN), POLIOVIRUS (INACTIVATED) TYPE 1 (MAHONEY STRAIN), HEPATITIS B SURFACE ANTIGEN (RDNA) ADSORBED ON ALUMINIUM HYDROXIDE [PRODUCED IN S. CEREVISIAE (STRAIN 2150-2-3) BY RDNA], ALUMINIUM HYDROXIDE
	DIPHTHERIA TOXOID, TETANUS TOXOID, HAEMOPHILUS TYPE B CONJUGATE VACCINE (TETANUS TOXOID CONJUGATE), PERTUSSIS TOXOID, POLIOVIRUS (INACTIVATED) TYPE 3 (SAUKETT STRAIN), POLIOVIRUS (INACTIVATED) TYPE 2 (MEF-1 STRAIN), PERTUSSIS TOXOID ADSORBED ON ALUMINIUM HYDROXIDE, HYDRATED, POLIOVIRUS (INACTIVATED) TYPE 1 (MAHONEY STRAIN), ALGELDRATE
	DIPHTHERIA TOXOID, TETANUS TOXOID, PERTUSSIS TOXOID, FILAMENTOUS HAEMAGGLUTININ
	DIPHTHERIA TOXOID, TETANUS TOXOID, PERTUSSIS TOXOID, PERTUSSIS FILAMENTOUS HAEMAGGLUTININ, PERTUSSIS PERTACTIN, ALUMINIUM HYDROXIDE
	DIPHTHERIA TOXOID, TETANUS TOXOID, PERTUSSIS TOXOID, PERTUSSIS PERTACTIN, POLIOVIRUS TYPE 1, POLIOVIRUS TYPE 2, POLIOVIRUS TYPE 3, FILAMENTOUS HAEMAGGLUTININ
	DIPHTHERIA TOXOID, TETANUS TOXOID, POLIOMYELITIS VIRUS (INACTIVATED) TYPE 1 PRODUCED IN VERO CELLS, POLIOMYELITIS VIRUS (INACTIVATED) TYPE 2 PRODUCED IN VERO CELLS, POLIOMYELITIS VIRUS (INACTIVATED) TYPE 3 PRODUCED IN VERO CELLS
	DIPHTHERIA TOXOID, TETANUS TOXOID, POLIOVIRUS TYPE 1 (INACTIVATED), POLIOVIRUS TYPE 2 (INACTIVATED), POLIOVIRUS TYPE 3 (INACTIVATED), ALUMINIUM HYDROXIDE
	HAEMOPHILUS INFLUENZAE TYPE B POLYSACCHARIDE (POLYRIBOSYLRIBITOL PHOSPHATE), DIPHTHERIA TOXOID, TETANUS TOXOID, POLIOVIRUS TYPE 1 (INACTIVATED), POLIOVIRUS TYPE 2 (INACTIVATED), POLIOVIRUS TYPE 3 (INACTIVATED), PERTUSSIS ANTIGEN
	HAEMOPHILUS TYPE B CONJUGATE VACCINE (TETANUS TOXOID CONJUGATE), HEPATITIS B SURFACE ANTIGEN (RDNA), POLIOVIRUS (INACTIVATED) TYPE 3 (SAUKETT

	STRAIN), PERTUSSIS FILAMENTOUS HAEMAGGLUTININ ADSORBED ON ALUMINIUM HYDROXIDE, HYDRATED, POLIOVIRUS (INACTIVATED) TYPE 2 (MEF-1 STRAIN), PERTUSSIS TOXOID ADSORBED ON ALUMINIUM HYDROXIDE, HYDRATED, TETANUS TOXOID ADSORBED ON ALUMINIUM HYDROXIDE, HYDRATED, POLIOVIRUS (INACTIVATED) TYPE 1 (MAHONEY STRAIN), DIPHTHERIA TOXOID ADSORBED ON ALUMINIUM HYDROXIDE, HYDRATED, ALGELDRATE, TETANUS PROTEIN
	HAEMOPHILUS TYPE B CONJUGATE VACCINE (TETANUS TOXOID CONJUGATE), HEPATITIS B SURFACE ANTIGEN, POLIOVIRUS (INACTIVATED) TYPE 3 (SAUKETT STRAIN), PERTUSSIS FILAMENTOUS HAEMAGGLUTININ ADSORBED ON ALUMINIUM HYDROXIDE, HYDRATED, POLIOVIRUS (INACTIVATED) TYPE 2 (MEF-1 STRAIN), PERTUSSIS TOXOID ADSORBED ON ALUMINIUM HYDROXIDE, HYDRATED, TETANUS TOXOID ADSORBED ON ALUMINIUM HYDROXIDE, HYDRATED, POLIOVIRUS (INACTIVATED) TYPE 1 (MAHONEY STRAIN), DIPHTHERIA TOXOID ADSORBED ON ALUMINIUM HYDROXIDE, HYDRATED, ALGELDRATE, TETANUS PROTEIN
	HAEMOPHILUS TYPE B CONJUGATE VACCINE (TETANUS TOXOID CONJUGATE), PERTUSSIS TOXOID ADSORBED ON ALUMINIUM HYDROXIDE, PERTUSSIS PERTACTIN ADSORBED ON ALUMINIUM HYDROXIDE, POLIOVIRUS (INACTIVATED) TYPE 3 (SAUKETT STRAIN), POLIOVIRUS (INACTIVATED) TYPE 2 (MEF-1 STRAIN), PERTUSSIS FILAMENTOUS HAEMAGGLUTININ ADSORBED ON ALUMINIUM HYDROXIDE, POLIOVIRUS (INACTIVATED) TYPE 1 (MAHONEY STRAIN), TETANUS TOXOID ADSORBED ON ALUMINIUM HYDROXIDE, DIPHTHERIA TOXOID ADSORBED ON ALUMINIUM HYDROXIDE, ALUMINIUM HYDROXIDE
	HAEMOPHILUS TYPE B CONJUGATE VACCINE (TETANUS TOXOID CONJUGATE), POLIOVIRUS (INACTIVATED) TYPE 3 (SAUKETT STRAIN), PERTUSSIS FILAMENTOUS HAEMAGGLUTININ ADSORBED ON ALUMINIUM HYDROXIDE, HYDRATED, POLIOVIRUS (INACTIVATED) TYPE 2 (MEF-1 STRAIN), PERTUSSIS TOXOID ADSORBED ON ALUMINIUM HYDROXIDE, HYDRATED, TETANUS TOXOID ADSORBED ON ALUMINIUM HYDROXIDE, HYDRATED, POLIOVIRUS (INACTIVATED) TYPE 1 (MAHONEY STRAIN), DIPHTHERIA TOXOID ADSORBED ON ALUMINIUM HYDROXIDE, HYDRATED, ALGELDRATE
	HAEMOPHILUS TYPE B CONJUGATE VACCINE (TETANUS TOXOID CONJUGATE), POLIOVIRUS (INACTIVATED) TYPE 3 (SAUKETT STRAIN), PERTUSSIS FILAMENTOUS HAEMAGGLUTININ ADSORBED ON ALUMINIUM HYDROXIDE, HYDRATED, POLIOVIRUS (INACTIVATED) TYPE 2 (MEF-1 STRAIN), PERTUSSIS TOXOID ADSORBED ON ALUMINIUM HYDROXIDE, HYDRATED, TETANUS TOXOID ADSORBED ON ALUMINIUM HYDROXIDE, HYDRATED, POLIOVIRUS (INACTIVATED) TYPE 1 (MAHONEY STRAIN), DIPHTHERIA TOXOID ADSORBED ON ALUMINIUM HYDROXIDE, HYDRATED, ALGELDRATE, TETANUS PROTEIN

	HAEMOPHILUS TYPE B CONJUGATE VACCINE (TETANUS TOXOID CONJUGATE), POLIOVIRUS TYPE 1 (INACTIVATED), POLIOVIRUS TYPE 2 (INACTIVATED), POLIOVIRUS TYPE 3 (INACTIVATED), PERTUSSIS FILAMENTOUS HAEMAGGLUTININ ADSORBED ON ALUMINIUM HYDROXIDE, HYDRATED, PERTUSSIS TOXOID ADSORBED ON ALUMINIUM HYDROXIDE, HYDRATED, TETANUS TOXOID ADSORBED ON ALUMINIUM HYDROXIDE, HYDRATED, DIPHTHERIA TOXOID ADSORBED ON ALUMINIUM HYDROXIDE, HYDRATED, ALGELDRATE
	PERTUSSIS TOXOID ADSORBED ON ALUMINIUM HYDROXIDE, HYDRATED, TETANUS TOXOID ADSORBED ON ALUMINIUM HYDROXIDE, HYDRATED, DIPHTHERIA TOXOID ADSORBED ON ALUMINIUM HYDROXIDE, HYDRATED, POLIOVIRUS (INACTIVATED) TYPE 2 (MEF-1 STRAIN) PRODUCED ON VERO CELLS, POLIOVIRUS (INACTIVATED) TYPE 3 (SAUKETT STRAIN) PRODUCED ON VERO CELLS, ALGELDRATE, POLIOVIRUS TYPE 1, INACTIVATED, STRAIN BRUNHILDE
	POLIOMYELITIS VIRUS (INACTIVATED) TYPE 1 PRODUCED IN VERO CELLS, POLIOMYELITIS VIRUS (INACTIVATED) TYPE 2 PRODUCED IN VERO CELLS, POLIOMYELITIS VIRUS (INACTIVATED) TYPE 3 PRODUCED IN VERO CELLS, TETANUS TOXOID ADSORBED ON ALUMINIUM HYDROXIDE, DIPHTHERIA TOXOID ADSORBED ON ALUMINIUM HYDROXIDE, ALUMINIUM HYDROXIDE
	POLIOVIRUS (INACTIVATED) TYPE 3 (SAUKETT STRAIN), POLIOVIRUS (INACTIVATED) TYPE 2 (MEF-1 STRAIN), PERTUSSIS TOXOID ADSORBED ON ALUMINIUM HYDROXIDE, HYDRATED, TETANUS TOXOID ADSORBED ON ALUMINIUM HYDROXIDE, HYDRATED, DIPHTHERIA TOXOID ADSORBED ON ALUMINIUM HYDROXIDE, HYDRATED, ALGELDRATE, POLIOVIRUS TYPE 1, INACTIVATED, STRAIN BRUNHILDE
	POLIOVIRUS (INACTIVATED) TYPE 3 (SAUKETT STRAIN), POLIOVIRUS (INACTIVATED) TYPE 2 (MEF-1 STRAIN), TETANUS TOXOID ADSORBED ON ALUMINIUM HYDROXIDE, HYDRATED, POLIOVIRUS (INACTIVATED) TYPE 1 (MAHONEY STRAIN), DIPHTHERIA TOXOID ADSORBED ON ALUMINIUM HYDROXIDE, HYDRATED, ALGELDRATE
	TETANUS TOXOID ADSORBED ON ALUMINIUM HYDROXIDE, HYDRATED, DIPHTHERIA TOXOID ADSORBED ON ALUMINIUM HYDROXIDE, HYDRATED
	TETANUS TOXOID, B/BRISBANE/60/2008-LIKE VIRUS, A/CALIFORNIA/7/2009 (H1N1) V REASS. X - 179 A, A/PERTH/16/2009 (H3N2) - LIKE STRAIN (A/VICTORIA/210/2009 REASS. NYMC X-187)
	TETANUS TOXOID, HAEMOPHILUS TYPE B CONJUGATE VACCINE (TETANUS TOXOID CONJUGATE)
	TETANUS TOXOID, HUMAN TETANUS IMMUNOGLOBULIN
	TETANUS TOXOID, SALMONELLA TYPHI BACTERIA (INACTIVATED)
